# Quantifying the impact of social activities on SARS-CoV-2 transmission using Google mobility reports

**DOI:** 10.1371/journal.pgph.0006368

**Published:** 2026-05-08

**Authors:** Felix Günther, Hilde Kjelgaard Brustad, Arnoldo Frigessi, Tom Britton

**Affiliations:** 1 Department of Mathematics, Stockholm University, Stockholm, Sweden; 2 Oslo Center for Biostatistics and Epidemiology, Oslo University Hospital, Oslo, Norway; 3 Oslo Center for Biostatistics and Epidemiology, University of Oslo, Oslo, Norway; 4 Integrate, University of Oslo, Oslo, Norway; Tohoku University Graduate School of Engineering School of Engineering: Tohoku Daigaku Daigakuin Kogaku Kenkyuka Kogakubu, JAPAN

## Abstract

We developed a new state-space model to investigate which social activities had biggest impact on the spread of SARS-CoV-2. Our analyses suggest that data from four categories of the Google community mobility reports capture an important share of transmission-relevant social activity. The analyses were based on reported hospitalizations and data on vaccinations, temperature, and virus strains. We applied our model to Sweden and Norway on a regional level over 17 months, and to the regions of Berlin and Bavaria in Germany over 10 months. Most results were shared for all three countries: Activity in the four social settings explained between 40–65% of all infections; *Public transport* appeared as an important setting for infections; and the transmission potential drops by around 40% during summer as compared to winter. However, the analyses for Germany differ in that *Retail and recreation* was the second activity setting dominating transmission whereas it was contacts at the *Workplace* in Norway and Sweden, showing how our model is able to adapt to specific cases. Transmissions not captured by the Google data may happen in other settings, in particular in households. In future pandemics, our method could be used in real time to guide more targeted intervention strategies.

## Introduction

How individuals move and interact in the society affects the transmissibility of an infectious disease. In mathematical modeling of infectious diseases, this is commonly reflected by defining the transmissibility as proportional to the product of the number of contacts between infectious and susceptible persons and the probability of infection per contact at a certain point in time. Depending on the pathogen, different modes of transmission exist. For respiratory viral diseases, like SARS-CoV-2, four central modes of infection are often considered: direct (physical) contact, indirect contact (via fomites), (large) droplets and (fine) aerosols [1]. Importance of these modes varies between pathogens and is often not entirely certain, remaining a point of scientific debate. From a theoretical perspective this implies that a somewhat abstract quantity is relevant to describe the spread of infectious diseases systematically: an actual average number of contacts that can lead to infections and includes all possible transmission routes.

Information on the (temporally and geographically varying) number or intensity of such contacts is of great importance for public health authorities and disease control. On the one hand, they can help to assess the potential for spread of a pathogen and thus the dangerousness of the situation; on the other hand, they allow to assess the effectiveness of any preventive measures taken. However, in practice, the (average) number of contacts relevant for transmission is unfortunately unknown and notoriously difficult to measure.

During the SARS-CoV-2 pandemic, different and partly novel data sources were used as proxies to measure (changes in) contact frequencies or, more generally, social activity within populations: contact surveys, mobility data collected, for example, by mobile phone providers, and other indicators resulting from repurposing routinely collected location data by various, often private actors. Such data sources have been used in various studies in the context of the SARS-CoV-2 pandemic focusing on different questions. For example, the Norwegian Institute of Public Health (FHI) has embedded Telenor mobile phone data in its meta-population models as a proxy for mobility between regions [2]. Badr et al. [3] used aggregated mobile phone data to create a social distance metric for each US county and investigated its correlation with the growth-rate of Covid-19 case counts. Mobile phone data have also been used to create detailed networks of how individuals in a population move between different points of interest and how they interact in different parts of the society [4,5]. Fritz et al. [6] used data from Facebook’s Data for Good program to investigate how regional differences in mobility patterns and friendship proximity affected the spread of COVID-19 in Germany. It is, however, oftentimes not entirely clear how well these data sources represent changes in relevant behaviour and contact frequencies, which is particularly important when being interested in directly using these indicators, and their variation in time and space, for disease surveillance. An important and publicly available data source on population behaviour during the SARS-CoV-2 pandemic were the Google community mobility reports (GCMR) [7]. This quantity was defined as the percentage increase or decrease in activity levels relative to a pre-pandemic baseline level in six different settings: grocery and pharmacy, retail and recreation, transit stations, parks, workplaces, and residential areas. The activity data are generated from mobile devices with a Google account where the location history setting is enabled. The GCMR data were collected and updated until 2022-10-15 and are freely accessible from Google in a fine-grained resolution in time and space. More details are given in the Materials and Methods section. These data were used in numerous studies investigating different aspects of SARS-CoV-2 spread. Cot et. al [8] combined the GCMR and Apple Maps Mobility Trends Reports (AMMTR) to quantify the impact of social distance on infection rates in European countries and in the US and to identify a time scale between the start of social distancing and the reduction in infection rates. Their results indicate a reduction of 20%-40% for European countries and 30%-70% in the US, during the second wave of the Covid-19 pandemic. A study by Nouvellet et al. [9], which combined a renewal process with a parametrization of the reproduction number as a function of GCMR and AMMTR, indicated a change in association between human behaviour and transmissibility over time. Basellini et al. [10] reduced the GCMR to a single mobility index by time and region in England and Wales through multilinear principal component analysis and linked estimates of excess mortality to this mobility index. A study by Yilmazkuday [11] used a difference-in-difference design with the different settings from the GCMR as a continuous treatment, to investigate its impact on change in COVID-19 cases and deaths for 130 countries. The results suggested that a 1% weekly increase in the GCMR residential setting led to about 70 less cases per week on average across all countries in the study. A 1% decrease in the work setting, transit setting or retail and recreation setting was estimated to give 18, 33 and 25 less cases, respectively. A study by Deng et. al [12] gives an overview of other studies using GCMR combined with different metapopulation models. While these studies are highly relevant, the application of the GCMR and the implementation of the data in the models for disease spread in these studies differ from our proposed method.

The aim of our work is to investigate to what extent the GCMR data are suitable to explain changes in transmission dynamics during the SARS-CoV-2 pandemic and whether the different activity categories of the GCMRs can be compared to make statements about which areas of social activity were particularly relevant for disease spread.

To achieve this, we have created a meta-population model, which we used to describe the disease spread in individual regions, and linked this model to a principled model of the association of GCMRs with contact frequencies among individuals in specific settings. Importantly, we carefully modeled the GCMR indicators, which refer to a pre-epidemic reference period and therefore need an accurate mathematical treatment, to make interpretation possible. This implementation of the GCMR, which takes into account how Google process the location data before making it public, is to our knowledge not previously done. We applied the model at a relatively fine spatial resolution for Norwegian and Swedish regions in the period from February 2020 to July 2021. The model allowed us to take into account changes in the probability of infection due to seasonality and the appearance of new virus variants, as well as the start of the vaccination campaigns in 2021. Our proposed model was flexible and adaptable, which we illustrate in a second analysis based on data from two different regions of Germany.

## Results

A central part of our work has been to develop a theoretically well-founded model in which the measurements of the GCMR can be brought into an interpretable relationship with the transmission dynamics of SARS-CoV-2. The model is described in detail in the Materials and Methods section. In the following, we briefly describe key aspects and assumptions of our model that are necessary to understand the results presented.

Our proposed model is an extension of a standard SIR-type dynamic transmission model that allows us to take into account general aspects that regulate the spread of an infectious disease - for example, the time-varying proportion of susceptible individuals in the population and the age dependent vaccination against SARS-CoV-2 in our study period. In such models, the transmissibility of a pathogen at a specific time point is represented by a transmission rate, which we express as a function of the GCMRs to model the relationship between SARS-CoV-2 transmission dynamics and human behaviour.

A central assumption of our model is that (transmission-relevant) contacts occur at different frequencies in different social settings as specified by the GCMRs, e.g., in the context of activities at work, in public transport, in retail and recreational contexts or in grocery shops and pharmacies. We assume that these contacts make an additive contribution to transmission and, importantly, that the activity per setting measured by the GCMRs is linearly related to the number of transmission-relevant contacts in the corresponding settings: if the GCMR activity level in a setting is reduced, e.g., by 50%, the model also assumes a reduction of transmission-relevant contacts in this setting by 50%. This linearity assumption appears plausible for four out of six the GCMR categories: *Retail and recreation*, *Grocery and pharmacy*, *Transit stations* and *Workplaces*. For the *Residential* setting, a linear relation between time spent at home and the average number of secondary infections appears implausible. Within a household contacts are so intense and persistent, and the number of household members is limited, that more time spent at home over a certain threshold likely has no significant impact on infections. For this reason, we have refrained from including the *Residential* setting explicitly in our model. The *Residential* setting is also strongly negatively correlated with the other settings and would hence introduce colinearities if kept in the analyses, as shown by the Condition Index [13] in the S1 Appendix. We also excluded the Park setting because very little transmission happened outdoor.

The central parameters estimated during model fit are region- and GCMR setting-specific coefficients $\phi_{k,r}$. They represent the average daily number of secondary infection(s) per infectious individual in region *r* in setting *k* at the pre-pandemic activity level. This is related to the product of two (unknown) quantities: the pre-pandemic activity level and the rate of infection-relevant contacts in each setting and region (see the Parameterisation of transmissibility parameter section). The parameters are therefore an estimate of how large the role of the specific social settings were for transmission at the *usual* (pre-pandemic) behaviour in each region.

Since the four considered GCMRs are unlikely to capture activity in all transmission-relevant social settings (especially since we do not explicitly consider activity and contacts in households, see above), we also estimate a region- and week-specific quantity $\nu_{week,r}$ that represents infections outside of settings captured by the GCMRs. By integrating the sum of the GCMR data for selected categories, weighted by the estimated coefficients, over time, we are able to quantify the overall share of infections explained by our model of the GCMR data, as well as the (retrospective) contribution of the different social settings to disease transmission (cf. the Materials and Methods section).

In addition to changes in population behaviour, our model also accounts for additional factors that can influence transmission dynamics, e.g., seasonality or changes in infectiousness due to mutations towards new virus variants. We assume that a change in temperature (as a proxy of seasonality) and the Alpha and Delta virus variants have a multiplicative effect on transmissibility, i.e., change the probability of transmission per contact.

### Model fit to hospitalization data

We fitted our model to weekly hospitalization counts of Swedish and Norwegian regions from February 21, 2020 to July 29, 2021, accounting for age-prioritized vaccination roll-out in the beginning of 2021. Estimation was performed in a Bayesian framework using Hamiltonian Monte Carlo in a custom implementation of the model in Stan [14] using the CmdStanR interface [15]. Detailed information on the definition of the region-level extended SIR model, the likelihood for the weekly hospitalization counts, as well as the fixed parameters and priors for the estimated parameters can be found in the Materials and Methods section. We fitted two separate models, one for joint modeling of the 11 Norwegian regions (*fylke*) and one for the 21 regions (*län)* of Sweden.

**Fig 1:**
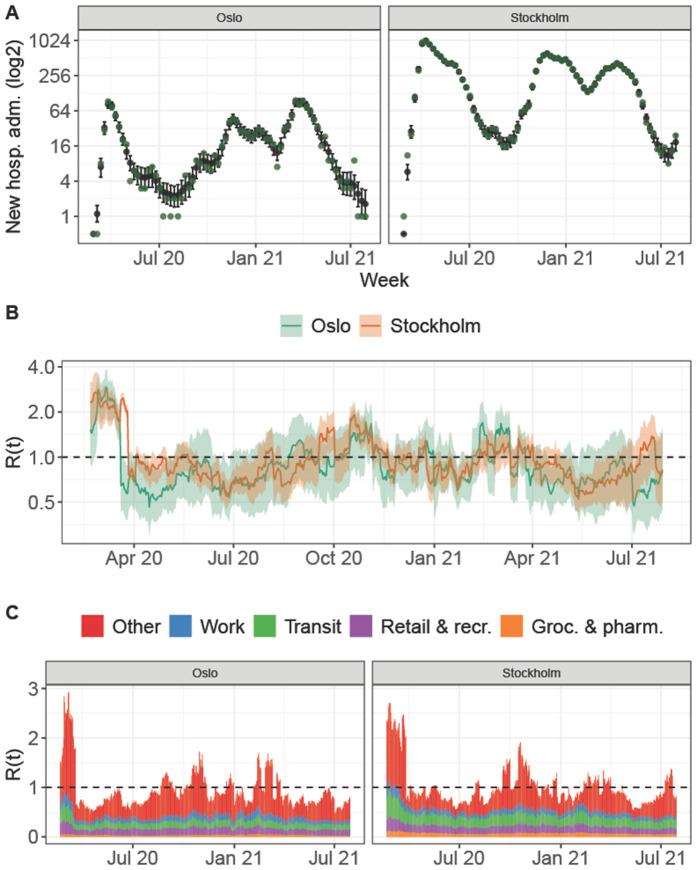
Model fit for two selected regions, Oslo and Stockholm. **Panel A** shows the weekly hospitalization counts: Oslo and Stockholm (green dots) as well as the model based expected hospitalization counts and associated 90%-credible interval. **Panel B** shows the estimated time-varying reproduction number, which is a function of the estimated ${\hat{\beta }}_{t,r}$ and corresponds to the expected number of secondary contacts per infected at each time-point and an associated 90%-credible interval. **Panel C** shows the model-based time-varying contribution of each transmission setting of the considered COVID-19 community mobility reports to these secondary infections.

Visual investigation of trace plots indicated convergence of the four independent MCMC chains per model to a joint posterior distribution, the convergence diagnostic $\hat{R}$ provided by Stan was smaller than 1.01 for all sampled parameters providing no evidence for convergence problems [16]. Fig 1 shows that our highly flexible model is able to capture the development of weekly hospitalization counts well, visualized based on the examples of Oslo in Norway and Stockholm in Sweden. Panel A illustrates the model fit, Panel B shows the estimated effective reproduction number and Panel C the contribution of the different GMCR categories over time. The flexibility of the model stems from the large amount of parameters that are estimated, in particular the region specific weekly number of secondary infection not captured in the GCMR setting, $\nu_{week(t),r}$. Fig. 26 - Fig. 31 in S1 Appendix provide similar plots for each Norwegian and Swedish region.

**Fig 2:**
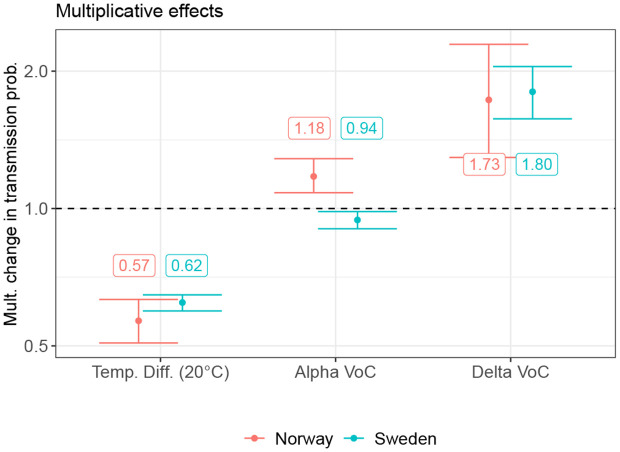
Multiplicative effects on transmission probability. Shown are the estimated posterior median and 90%-credible intervals for the multiplicative effects of VoC’s and a 20-degree temperature difference on the transmissibility for the Norwegian (red) and Swedish (blue) regions, respectively.

In addition to changes in contact frequencies represented via the additive model for the GCMR, our model of SARS-CoV-2 transmissibility accounts for multiplicative changes in infection risk per contact associated with the Alpha and Delta variants of concern (VoC) and seasonal effects. We assume that these multiplicative effects are the same in all regions considered in the respective model (Sweden or Norway), and our estimates from the data of the Norwegian and Swedish regions are quite similar, as shown in Fig 2. We estimate that an increase in temperature of 20 degree Celsius (corresponding approximately to the difference between the average temperature of the coldest winter and warmest summer month) leads to a reduction in transmissibility by around 40%. Note that the median difference between the mean temperature in the warmest and coldest month of the year per region is 20.4 degrees in our observation period. For the Alpha VoC, we estimated an increase in transmissibility compared to the wild-type of around 20% based on data of the Norwegian regions but found no clear evidence for an increase in transmissibility in the Swedish data. For the Delta VoC, we estimated approximately a doubling of transmissibility compared to the wild-type in both Norway and Sweden, albeit with substantial uncertainty.

### Activity at Transit stations and Workplaces capture most important settings of infection in Norway and Sweden

**Fig 3:**
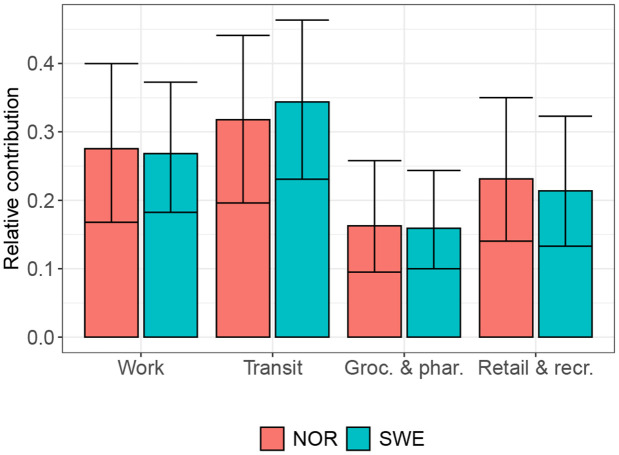
Population-weighted relative contribution of the community mobility reports at BL activity. Shown are the posterior median and 90%-credible intervals for the population-weighted relative contribution of each GCMR setting at baseline activity for Norway and Sweden.

Based on the estimated parameters $\phi_{k,r}$ from our model, contacts in settings captured by the Transit and Workplaces categories lead to most infections at the *usual* pre-pandemic activity: the point estimate for the Transit was highest in 7 out of 11 Norwegian regions and in 9 out of 22 Swedish regions. In the remaining 4 regions in Norway and in 11 of the 12 remaining regions in Sweden the Workplace category was most important (see Table 3 in S1 Appendix). Fig 3 shows the population-weighted average of the relative share of secondary infections assigned to each setting at baseline activity. If activity stayed unchanged, we estimate most infectious contacts captured by activity at the Transit setting, followed by Workplaces, Retail & recreation, and lastly Grocery & pharmacies for Norway and Sweden. The posterior probability that the population-weighted mean of the Transit category is largest among the four categories is 0.55 for Norway and 0.68 for Sweden. Furthermore, we estimated a posterior probability of 0.50 and 0.62 that the Transit and Workplace categories make up the settings with the largest contribution to transmission among the six two-setting combinations at baseline activity.

### Model attributed 60% of all infections to contacts in four GCMR settings for Sweden and 39% for Norway

Taking into account observed changes in activity in the GCMR categories, we derived the total share of infections that were attributed to contacts in settings captured by the four GCMR categories per country and for each county (see the Materials and Methods section). Aggregated over the whole time period and all counties, our model allocated 60.0% (90%-CI: 57.1-62.5%) of all infections in Sweden and 38.7% (33.2 - 43.8%) of all infections in Norway to settings captured by the GCMRs (Fig. 32 in S1 Appendix). The remaining, unexplained, infectious contacts were accounted for by the region-specific weekly parameters $\nu_{week(t),r}$. Among the GCMR categories, settings captured by the Transit category still played the largest role when taking into account changes in activity over time (with 10.9% of all infections in Norway and 18.9% in Sweden). Differences in the share of infections between the categories were, however, less pronounced compared to the expected share of infections at baseline activity: the relative contribution of contacts captured by the Work category decreased, and the importance of contacts captured by the Retail and recreation category increased (Table 5 in S1 Appendix).

When investigating how much of the SARS-CoV-2 transmission dynamics was explained by the GCMRs in each county overall and per category, we found rather large differences. The mean share of daily infectious contacts in settings captured by the GCMRs varied between 27.5% and 73.3%, the total share of infections assigned to the settings captured by the GCMRs was in a similar range, albeit slightly smaller for most regions (Fig. 32, Table 6 and Table 7 in S1 Appendix).

The GCMRs captured a larger share of infectious contacts in Swedish compared to Norwegian counties. We also observed a tendency towards a larger explanatory power for regions with higher population density. However, this correlation is rather weak and to a large extent driven by the capitals, Oslo and Stockholm, with high population density (Fig. 34 in S1 Appendix).

### German regions

Our proposed model to describe the relationship between GCMRs and SARS-CoV-2 transmission rate, is not specific to Norway and Sweden, nor to the time period analyzed above. We estimated the model for two different regions from Germany, Bavaria and Berlin, in a shorter period (Feb 2020 - Dec 2020) based on publicly available data. For this period, no relevant virus variants or vaccination activity had to be considered (for more details, see the Materials and Methods section).

**Fig 4:**
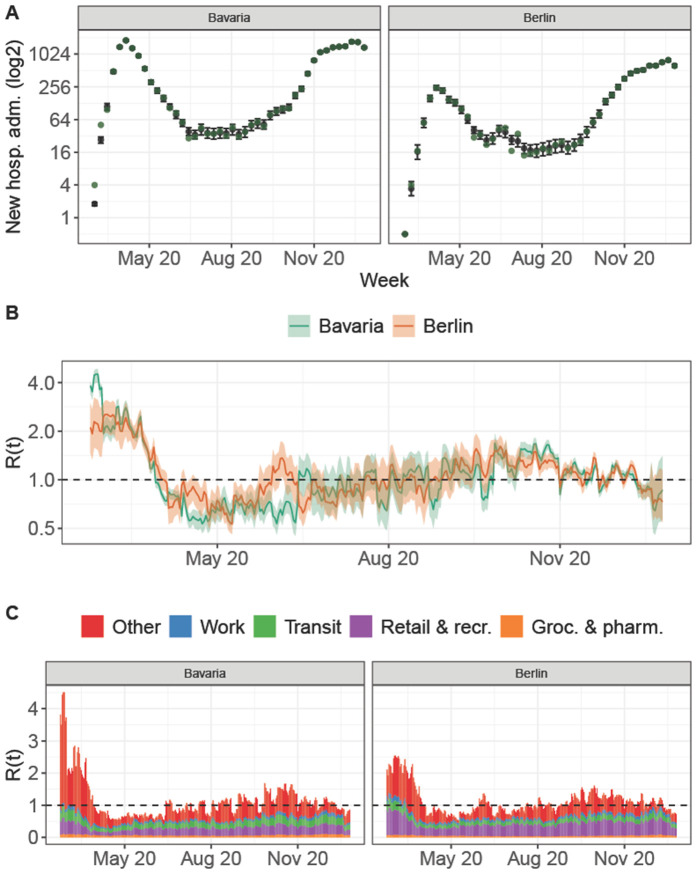
Model fit for the two German regions, Bavaria and Berlin. **Panel A** shows the weekly hospitalization counts: Bavaria and Berlin (green dots) as well as the model based expected hospitalization counts and associated 90%-credible interval. **Panel B** shows the estimated time-varying reproduction number, which is a function of the estimated ${\hat{\beta }}_{t,r}$ and corresponds to the expected number of secondary contacts per infected at each time-point and an associated 90%-credible interval. **Panel C** shows the model-based time-varying contribution of each transmission setting of the considered COVID-19 community mobility reports to these secondary infections.

**Fig 5:**
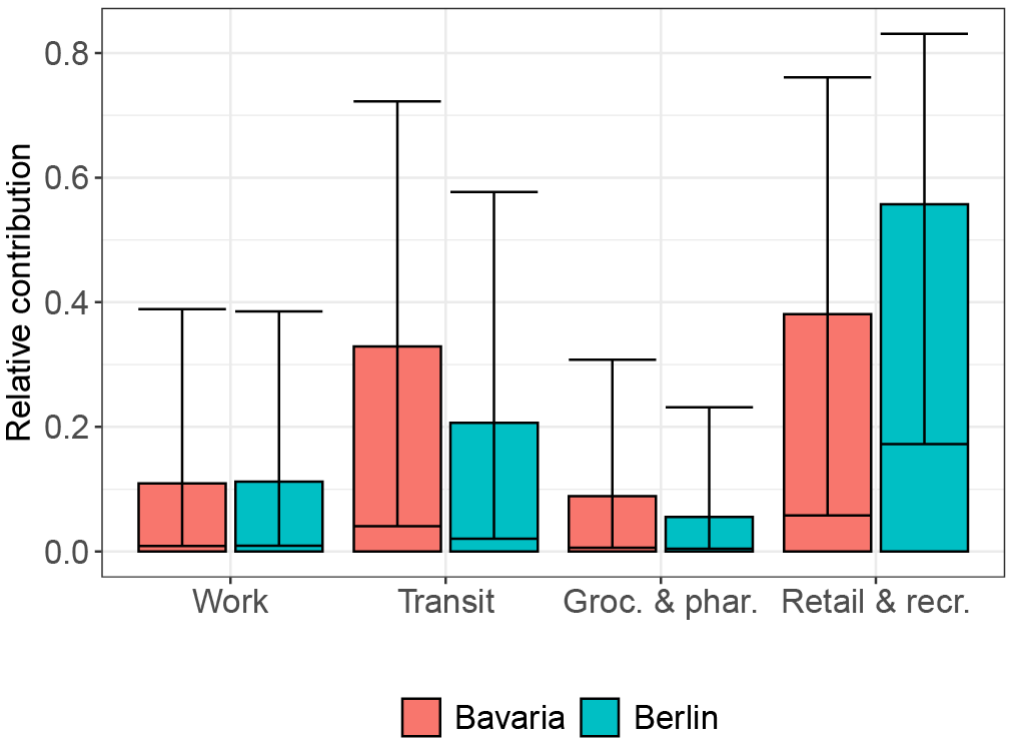
Relative contribution of the community mobility reports at BL activity, Bavaria and Berlin. Shown are the posterior median and 90%-credible intervals for the relative contribution of GCMR setting to the transmissibility at baseline activity, for the two analyzed German regions Bavaria and Berlin.

Fig 4 shows the model fit, estimated effective reproduction number and the contribution of the different GCMR categories for Bavaria and Berlin. Compared to the results in the Norwegian and Swedish regions, the GCMR activity categories captured a relatively large share of infectious contacts (64.5% in Bavaria, 69.7% in Berlin on a daily average). During the observation period, 65.2% (90%-CI: 58.7-69.9%) of all infections in Berlin and Bavaria were allocated to contacts in settings captured by the GCMRs. The multiplicative effect of the temperature on the disease transmission in Germany had a similar order of magnitude compared to Sweden and Norway: a 20 degree increase in temperature reduced the model-based transmission probability per contact by factor 0.50 (90%-CI: 0.44-0.59). The largest difference compared to the results in Sweden and Norway was the model-based relative contribution of the different GCMR categories to transmission, both at baseline activity and aggregated over the analysis period. In both German regions, and in particular Berlin, we estimated that activity in settings captured by the Retail & recreation category contributed most to infectious contacts at baseline activity, but also accounting for changes in activity over the observation period. It is followed by the Transit category, which instead was most important for Sweden and Norway (Fig. 5 in current text and Table 9 in S1 Appendix).

### Sensitivity analysis for Germany

Our investigation of collinearity between the categories of the GCMR showed that for the German regions, the *Transit stations* setting was highly correlated with both the *Retail and recreation* setting and the *Workplaces* setting (Fig. 9 in S1 Appendix). Further investigation of multicollinearity between the categories, by the use of Condition Index (ref to litteratur), showed moderate to strong multicollinearity between the four categories *Grocery and Pharmacy*, *Retail and Recreation*, *Transit stations* and *Workplaces*. Excluding the *Transit stations* setting reduced this multicollinearity for both regions (Fig. 13 in S1 Appendix).

We performed a sensitivity analysis for the German regions, by removing the *Transit station* setting from the analysis. The model fit to hospitalizations remain good, see Fig 35 in S1 Appendix. After removing the *Transit stations* setting, the relative contribution of each setting to the transmissibility at baseline activity increases, with the biggest increase for the *Retail and recreation* setting (Table 11 and Fig. 36 in S1 Appendix). The estimated coefficients $\phi_{k,r}$ increases for each setting when removing *Transit stations* (Table 10 in S1 Appendix). The estimated share of the secondary infections attributed GCMR categories increases when removing the transit setting (Table 12 and Fig. 37 in S1 Appendix). Even though the contribution of each setting to different effect measures increases when removing the *Transit stations* setting, the differences are not big and the credible intervals do not indicate any significant difference in the results of the main analysis and the sensitivity analysis.

## Discussion

We proposed a new model to quantify the contribution of population behaviour in different social settings on the spread of SARS-CoV-2, based on the Google Community Mobility Reports. In our state-space model for disease spread over time, the transmissibility parameter was expressed as a function of the GCMRs in four social settings, as well as of daily temperature and the proportion of different virus variants of concern. Importantly, the model was constructed to account for the precise definition of the GCMR indicators, so that each setting was modeled to have an additive contribution to the transmissibility. The rationale for this was that an increase/decrease in time spent in a specific social setting leads to an increase/decrease in transmission-relevant contacts in this setting, but does not affect transmissions in other settings. The temperature and virus variants on the other hand, were modeled to have a multiplicative effect on the transmissibility, thus affecting all types of transmission. Remaining changes in transmission dynamics were captured by a region- and week-specific random effect. This model allowed us to evaluate how well the GCMRs capture changes in disease dynamics during the SARS-CoV-2 pandemic, which settings of contacts appeared to be most relevant for transmission, as well as how disease transmission was related to temperature (as a proxy for seasonality) and virus variants. We applied the model to data from all Norwegian and Swedish regions and to two large German regions. This showed that the model is flexible and will be easy to apply to new situations in the future, when it can be used during an ongoing epidemic as opposed to the current retrospective analysis.

The results of our analyses suggest that the GCMR data reflect changes in population behaviour that were directly related to the dynamics of the spread of SARS-CoV-2. Based on our model, for Sweden 60% of infections were attributed to social settings that were captured by the four GCMR on Workplaces, Transit stations, Groceries & Pharmacies, and Retail & Recreation, and 39% for Norway. Both percentages, and similarly for the German data, were interestingly high. The remaining infections cannot be explained by changes in population activity measured by the four GMCRs. The household setting is most likely a major additional infection setting.

We estimate that at regular, pre-pandemic activity-level contacts captured by the Transit station and Workplace category had the highest contribution to transmissibility among the four settings for both Norway and Sweden. Also when considering the observed changes in activity over our observation period, contacts captured by the in the Transit category played a large role. It was, however, interesting to see that the (relative) importance of activity at Workplaces decreased, while the importance of Retail and Recreation increased. This lets us hypothesize, that these modes of social activity play a large role for disease transmission and reduction in activity at these settings can substantially reduce disease spread.

In our model, we accounted for seasonality and presence of virus variants by estimating multiplicative effects on the probability of infection upon contact between a susceptible and an infectious individual. For the VoCs, we also assumed an increase in hospitalization risk among infected by factor 1.9. We estimated a substantial change in transmissibility due to seasonality (transmissibility reduced by ∼ 40% in summer compared to winter) and increased transmissibility due to the the Delta VoC compared to the wild-type. In the Norwegian model, we also estimated a slight increase in transmissibility for the Alpha VoC. These results were in line with the existing literature and the effect of seasonality was estimated consistently for Sweden and Norway.

Please note that the estimated effects are based on actual changes in behaviour as measured by the Google GCMR data, as opposed to other analyses estimating effects of introduced restrictions, whether or not these were followed, such as the Oxford Stringency index [17]. Note also that the similarity in effect estimates between Norway and Sweden by no means imply that the actual transmissibility over time were the same in the two countries, or even in regions within a country. For instance, as can be seen in panel B of Fig 1, Norwegians reduced their overall social activity about 10 days earlier than Sweden, with the effect that transmissibility (measured by *R*(*t*)) dropped below 1 about 10 days before the drop in Sweden. And, relating to stringency indices, Norway had many more restrictions than Sweden (who mainly had recommendations) but as can be seen in the same panel, the overall social activity reduction were fairly similar during most parts of the pandemic.

We also fitted the model to two German regions of similar size as countries Norway and Sweden, Bavaria and Berlin, for the first 10 months of the pandemic. In these regions and time period, the GCMRs explained a slightly larger share of infections compared to our analyses for Sweden and particularly Norway. Just like for Norway and Sweden, the Transit station setting had a high contribution to transmission, and Groceries and pharmacies played the lowest role for transmission. The seasonality effect was estimated similarly for the German regions as in Norway and Sweden. However, in contrast to the Swedish and Norwegian results, the analyses of the German regions found that at pre-pandemic activity levels, the Retail & recreation setting played the largest role for transmission and contacts captured by the Workplace category had only a smaller contribution. In our model, the importance of each category can be factorized into two (unknown) components: the (absolute) degree of activity and corresponding amount of social contacts in a pre-pandemic social context and the transmission rate per contact in the category. It is quite conceivable that, particularly in Berlin and the urban centers of Bavaria, the baseline level of activity in the retail and recreational sector is substantially higher than in many of the more rural regions of the Nordic countries, thus explaining the higher importance of Retail & recreation in the German regions. It would be interesting to study further in detail what drives these such differences, comparing, e.g., the social behaviour, the organisation of labour, the mobility systems, and the demographic profiles in these regions in designated studies. Of course, differences in the in the GCMR data and the different analysis periods could also partly explain the observed differences in the results.

The proposed model is of course a simplification of reality and has some limitations: First, we assumed that changes in the considered GCMR settings were linearly related to the effective number of transmission-relevant contacts. This means for example that we assume that if GCMR-measured activity at workplaces is reduced by, say, 20%, then the number of transmission-relevant contacts in workplaces is reduced by 20% as well. Furthermore, we assumed that this linear relationship was constant over time, ignoring potential behavioural changes in specific settings, for example due to increased distancing between people or introduction of face masks. We think that both assumptions are plausible at a first order. Also, we assumed that effects of seasonality and virus variants change transmission risk in a multiplicative way in all social settings. One could argue that the effect of seasonality could be setting-specific, the infection risk at workplaces might be changed differently as in Public transport.

In population-wide dynamic transmission models, it is common to consider the age structure in a population and to adjust for known patterns in age-specific contact frequencies (see, e.g., [18]). The core of our work is to model time-varying contact frequencies during the SARS-CoV-2 pandemic using the GCMRs. Since these are only available at the population level (and not in specific age groups), it is not possible to fully account for age and the associated contact structure in this analysis. The compromise of our model corresponds to an assumption that contacts in the population occur randomly (and independently of the age of the individuals involved, i.e., homogeneous mixing), and the frequency of these contacts varies in time and between regions. Nevertheless, we included an age structure to take into account the important aspect of age prioritization at the start of the COVID-19 vaccination campaign. Also we did not explicitly account for transmission between different regions in the dynamic transmission model, but modeled transmission separately within each region. Since the population size in the different regions is relatively large (it varies between 130,000 and 2.4 million, except for the Swedish island Gotland) and spatial dynamics were not the focus of our work, this seems to be a reasonable simplification, also because we did not have detailed information on inter-regional mobility and its changes during the analysed period. Our model assumes an all-or-nothing effect of vaccination and a maintained full protection of vaccination during the modeling period. A more realistic modeling of vaccination would be to implement a partial immunity upon vaccination and a gradual waning over time. However, our study period is possibly short enough so that waning of vaccine-induced immunity did not play a major role and then this assumption would not introduce a major error.

Also, we specified several aspects of the model (e.g., generation time, duration of infectiousness, increased hospitalization risk due to VoCs) based on consolidated literature. Wrong assumptions on these parameters might bias our results. In our proposted model, we have not included explicitly term for observation error and process errors, as in many other papers [19], however, it could be interesting to implement this in the future.

A final concern is about the possible measurement errors of the GCMRs, which may not accurately enough capture changes in the amount of the social interactions between individuals in the specific categories of interest defined in the GCMRs. This may limit the causal interpretability of our results related to the (relative) importance of contacts in the different settings.

Overall, our analysis found a clear association between the publicly available GCMRs and SARS-CoV-2 infection dynamics in Norway, Sweden and two German regions. This indicates, that such data can be useful for a real-time assessment of the level of relevant population activity in times of an epidemic/pandemic. However, definition and structure of GCMRs (using relative changes) does not facilitate a straightforward utilization in mathematical models of disease transmission. For this, it requires rather strong assumptions, a complex model and estimation of many parameters. Also, we observed variability in the association of different GCMR categories with disease dynamics for the different regions in our model. Both of these aspects can limit the use of data in real-time analyses. It is conceivable that more direct measurements of (absolute) contact frequencies would have a bigger explanatory power with respect to actual transmission dynamics, and the real-time generation of such data would therefore be desirable for the future.

## Materials and methods

For Norway and Sweden, all computations were run for a period of about 17 months, from February 21, 2020 to July 29, 2021 (75 weeks). The spatial resolution of the model was by fylke in Norway and län in Sweden. In total 32 regions were included in the analysis, 11 in Norway and 21 in Sweden. Lower spatial resolution, i.e., municipality level, resulted in higher degree of missing GMCR data due to anonymity issues and in lack of hospitalization data.

### Google community mobility reports (GCMR)

Google provides a freely available data source, the Google COVID-19 Community Mobility Reports, a daily, region specific, activity level in different social settings [7]. These data are based on tracking Google users, who have “Location History” enabled on their mobile devices. Geographic locations are classified into six settings: Grocery and pharmacy, Retail and recreation, Transit stations, Parks, Workplaces, and Residential areas. For the first four settings, the GCMR index measures the percentage increase/decrease in the daily number of visits at locations classified to each setting, relative to a pre-pandemic baseline level. The baseline level, one for each day of the week, is the average number of visits at the locations and setting, measured over 5 consecutive weeks pre-pandemic, between Jan 3 and Feb 6, 2020. The daily number of visits in a setting is then divided by the baseline number in that setting of the corresponding weekday, and scaled so that 0 corresponds to the baseline level, 100 corresponds to a doubling in the number of visitors and, e.g., −50 corresponds to a reduction in visitors by 50%. The residential and workplace settings measure the length of stay (in minutes) during a 24 hour period rather than number of visits and are otherwise defined analogously. An unspecified, small amount of noise is added to the data before computation of the baseline values and the daily counts to meet anonymity requirements [20].

Let $x_{t,r}^{k}$ denote the Google covariate used in our model, at day *t* in region *r* in setting *k*. We have rescaled the da*t*a provided by Google, such that $x_{t,r}^{k}=-0.5$, corresponds to a 50% reduction in activity level, $x_{t,r}^{k}=0$ corresponds to the same activity level as at baseline and $x_{t,r}^{k}=1$ corresponds to a 100% increase in activity. Furthermore we used a (right-aligned) 7-day moving average of the data for our model to deal with the inherent weekday fluctuations in the GCMR data. Fig. 1 in S1 Appendix shows the mean GCMR and variation across regions for Norway and Sweden, for the different settings.

### Temperature

Daily average temperature for Norway was acquired from the Norwegian Meteorological Institute Frost API, while the Swedish temperature data was acquired through the Open Data Meteorological Observations API provided by Swedish Meteorological and Hydrological Institute. For each region a weather station close to the most dense populated area was selected. The full list of stations selected is given in Table 1 in S1 Appendix.

### Variants of concern data

The World Health Organization (WHO) declared the Alpha variant (pango lineage B.1.1.7) a Variant of Concern (VoC) on December 18, 2020. On May 11, 2021, WHO classified Delta (pango lineage B.1.617.2) as a VoC. Ideally we want to know the proportion of the true number of cases that are of the different variants. This is unknown because the true incidence in the population is unknown and not every case was sequenced. Instead, we approximate the true proportion by the proportion of the sequenced/screened cases, denoted $x_{t,r}^{\alpha }$ and $x_{t,r}^{\delta }$ for the Alpha and Delta variants, respectively. The share of Alpha and Delta variants over time was only available on a national level in a weekly temporal resolution. The same time series were therefore used for all regions within a country. Fig. 20 in S1 Appendix shows the data. For Norway, the data was acquired manually from the weekly Covid-19 reports of the National Institute of Public Health [21] and the Swedish data was acquired from the web pages of Public Health Agency of Sweden [22].

### Vaccination data

Vaccination data is given as daily number of new individuals who have received the second vaccine dose, by region and by age group, denoted $v_{t,r}^{i}$ for day *t*, region *r* and age group *i*. See Table 2 in S1 Appendix for age groups. Data for Norway was downloaded from the GitHub of The Norwegian Ins*t*itute of Public Health [23] and data for Sweden was provided from the Public Health Agency of Sweden upon request. The vaccine data were not updated every day of the week. We therefore assume a linear increase in the cumulative number of vaccinated individuals at days with missing data. See Fig. 22 - Fig. 24 in S1 Appendix.

### Model for disease spread

We model the spread of disease over time in a region by a discrete time deterministic meta-population model, an age-structured SIR model (see Fig. 25 in S1 Appendix). The population in region *r* at day *t* and age-group *i* is divided into susceptible individuals $S_{t,r}^{i}$, infectious individuals $I_{t,r}^{i}$, individuals recovered from infection $R_{t,r}^{i}$ and vaccinated individuals *V*_*t*,*r*_. Transitions between the compartments are given by the following set of equations:


$$\begin{array}{l} S_{t+1,r}^{i}-S_{t,r}^{i}=-\frac{\beta_{t,r}}{N_{r}}I_{t,r}S_{t,r}^{i}-\frac{n_{\text{imp}}}{10^{5}}S_{t,r}^{i}-p_{v}v_{t,r}^{i}\frac{S_{t,r}^{i}}{R_{t,r}^{i}+S_{t,r}^{i}}\\ I_{t+1,r}^{i}-I_{t,r}^{i}=\frac{\beta_{t,r}}{N_{r}}I_{t,r}S_{t,r}^{i}+\frac{n_{\text{imp}}}{10^{5}}S_{t,r}^{i}-\gamma I_{t,r}^{i}\\ R_{t+1,r}^{i}-R_{t,r}^{i}=\gamma I_{t,r}^{i}-p_{v}v_{t,r}^{i}\frac{R_{t,r}^{i}}{R_{t,r}^{i}+S_{t,r}^{i}}\\ V_{t+1,r}-V_{t,r}=p_{v}\sum_{i=1}^{G}v_{t,r}^{i} \end{array}$$

where *N*_*r*_ is the population size of region *r*. Infectious individuals at day *t*, $I_{t,r}=\sum_{i=1}^{G}I_{t,r}^{i}$, interact homogeneously with *t*he susceptible individuals in each age group. The transmissibility parameter $\beta_{t,r}$, is defined as the average number of infectious contacts an infected individual has at day *t* in a fully susceptible population. We model this parameter as a function of region specific covariates and associated parameters are estimated. In addition *t*o the susceptible individuals infected by individuals from the infectious compartments, we assume that *n*_*imp*_ per 100 000 susceptible individuals in each age group are infected each day and moved to their respective infectious compartment, to mimic importation of cases. In our main analysis, we specified *n*_*imp*_ = 0.5. Infectious individuals in age group *i* are moved to the recovered naturally compartment $R_{t,r}^{i}$ with a rate of 1/γ days.

The modeling period extends over a time period where vaccination was implemented. Age prioritized vaccination changes the age composition of the infected individuals, which then changes the risk of hospitalization. The likelihood of hospitalization at time *t* depends on the probability that a randomly selected susceptible individual belongs to a specific age group, hence, we need to keep track of the number of susceptible individuals in each age group. This motivates the age structure of the meta-population model.

Vaccinated individuals are assumed to either become fully immune, hence not contributing to further transmission of the disease, or else, with probability *p*_*v*_ the vaccine has no effect. The daily number of new individuals with full immunity from vaccine, $p_{v}v_{t,r}^{i}$, are removed from the susceptible and naturally recovered compartments, according to the relative size of $S_{t,r}^{i}$ and $R_{t,r}^{i}$. Immunity is assumed to remain over the study period.

### Parameterisation of transmissibility parameter

We want to relate the transmissibility parameter $\beta_{t,r}$ to multiple covariates: the GCMRs, temperature (as a proxy for seasonality) and share of specific virus variants. The different GMCR settings are assumed to have an additive effect on the transmissibility, while the temperature and the share of circulating virus variants are assumed to have a multiplicative effect.

The additive part of the transmissibility, $\beta_{t,r}^{add}$, as a function of the GCMRs, is derived as follows. Assume that the average number of secondary infections of an infectious individual occur in *K* different settings, e.g., at work, public transport etc. For setting *k*, we define the average number of secondary infection at day *t* in region *r*, $\beta_{t,r}^{k}$, as the product between *t*he average number of contacts in that setting, $c_{t,r}^{k}$, and the probability that a contact results in an infection, $p_{r,k}^{inf}$. The number of contacts in each setting is unknown. By assuming a linear association between the GCMRs and the number of contacts in a setting, the number of secondary infections in setting *k* becomes


$$\begin{array}{rcl} \beta_{t,r}^{k} & = & c_{t,r}^{k}\cdot p_{r,k}^{\text{inf}}\\ & = & (1+x_{t,r}^{k})\cdot c_{\mathrm{BL},r}^{k}\cdot p_{r,k}^{\text{inf}}\\ & = & (1+x_{t,r}^{k})\cdot \phi_{k,r} \end{array}$$

where $c_{BL,r}^{k}$ is the baseline level (pre-pandemic) number of contacts in setting *k* in region *r*. The parameter $\phi_{k,r}$ can be interpreted as the average daily secondary infections per infectious individual in region *r*, in setting *k* at the baseline activity level, i.e., pre-pandemic level when $x_{t,r}^{k}=0$.

The number of secondary infections caused by an infectious individual during a day is then the sum of secondary infections in the different settings. In addition to the *K* settings, we include daily secondary infections happening outside of the considered GCMR transmission settings by adding a weekly region specific parameter, $\nu_{week(t),r}$ (e.g., household transmission), assumed to be a priori i.i.d. between weeks and across regions based on a half-normal prior with region-specific standard deviation (cf. Implementation). The additive part of the transmissibility at day *t* in region *r* is then


$$\beta_{t,r}^{add}=\nu_{week(t),r}+\sum_{k=1}^{K}(1+x_{t,r}^{k})\cdot \phi_{k,r}.$$

In our model fitting, we included *K* = 4 settings from the GCMR covariates: Grocery and pharmacy, Retail and recreation, Public transport and Workplace. We excluded the Park (not adding to transmission) and Home settings as the assumption of a linear increase in infectious contacts with increasing activity seems less plausible for these settings, and home setting having strong (negative correlation with the other factors.

Transmission dynamics are not only related to changes in contact frequencies. Seasonality and virus variants alter transmissiblity of a pathogen substantially. We use region-specific daily mean temperature as a proxy for seasonality and assume that the seasonality as well as the Alpha and Delta virus variants act multiplicatively on the transmissibility by changing the probability of infection upon contact. The multiplicative part of the transmissibility is modeled as


$$\beta_{t,r}^{mlt}=e^{\phi_{temp}x_{t,r}^{temp}+\phi_{\alpha }x_{t,r}^{\alpha }+\phi_{\delta }x_{t,r}^{\delta }},$$

where $x_{t,r}^{temp}$ is the temperature difference at day *t* compared to the first day of *t*he analysis in region *r*, and $x_{t,r}^{\alpha }$ and $x_{t,r}^{\delta }$ represent the fraction of individuals infected by the two variants of concern. The parameters $\phi_{temp}$, $\phi_{\alpha }$ and $\phi_{\delta }$ are assumed constant over region and in time. The multiplicative change in transmission probability for a one-degree increase in temperature is then $e^{\phi_{temp}}$, and $e^{\phi_{\alpha }}$ or $e^{\phi_{\delta }}$ correspond to a multiplicative increase in transmissibility for the Alpha or Delta VoC compared to the wild-type. To characterize the total effect of seasonality we focus on the multiplicative change for a difference in temperature of 20 degrees, $e^{20\times \phi_{temp}}$, which corresponds to the median temperature difference between the average temperature in the warmest and coldest months of the year for the Norwegian and Swedish regions.

Combining the additive and multiplicative parts $\beta_{t,r}^{add}$ and $\beta_{t,r}^{mlt}$, we get the final parameterization of the transmissibility


$$\beta_{t,r}=(\nu_{week(t),r}+\sum_{k=1}^{K}(1+x_{t,r}^{k})\cdot \phi_{k,r})\times \;e^{\phi_{temp}x_{t,r}^{temp}+\phi_{\alpha }x_{t,r}^{\alpha }+\phi_{\delta }x_{t,r}^{\delta }}.$$

The parameters that we need to estimate in the above parameterization of $\beta_{t,r}$ are $\nu_{week(t),r}$ (for 75 week and each region), $\phi_{k,r}$ (for each region and *K* = 4 behaviour settings), $\phi_{temp}$, $\phi_{\alpha }$ and $\phi_{\delta }$.

### Interpretation of model parameters and derived quantities

We focus on two aspects when interpreting the estimated parameters $\phi_{k,r}$ for the GCMRs. First, the proportion of variation in the additive part of transmissibility, which is explained by the linear model in combination with the observed change in the community mobility report data. For this purpose, we can quantify per region the mean share of mobility reports in $\beta_{t,r}^{add}$ over time: $(1/T)\times \sum_{t=1}^{T}[(\sum_{k=1}^{K}(1+x_{t,r}^{k})\cdot \phi_{k,r})/\beta_{t,r}^{add}]=(1/T)\sum_{t=1}^{T}\pi_{t,r}^{MR}$. We refer to this quantity as the mean share of daily infectious contacts in settings captured by the community mobility reports. A similar summary statistic also takes into account the time-varying number of susceptible and infected individuals: $\left(\sum_{t=1}^{T}\pi_{t,r}^{MR}\beta_{t,r}S_{t,r}I_{t,r}N_{r}^{-1})/(\sum_{t=1}^{T}\beta_{t,r}S_{t,r}I_{t,r}N_{r}^{-1}\right)$. It represents the overall share of secondary infections in settings captured by the community mobility reports model over the whole observation period. These calculations can be performed for all *K* = 4 categories of the GCMRs combined or for each category separately. A second aspect is related to which of the four considered community mobility reports captures population behavior that appears to be most relevant for disease transmission. This can be addressed by investigating the relative size of the coefficients $\phi_{k,r}$ for the *K* = 4 categories: $\phi_{k,r}^{rel}=\phi_{k,r}/\sum_{k}\phi_{k,r}$. We perform posterior inference for these parameters $\phi^{rel}$ per region and combine the region-specific estimates based on a population-weighted mean over all regions of a country.

### Fitting the model

We fit the model to weekly number of new hospital admissions due to SARS-CoV-2 infection in each region. The number of hospital admission in week *w* is assumed to follow a Poisson distribution, with expectation dependent on daily new number of infected individuals, distribution of time between infection and hospitalization and risk of hospitalization given infection. Due to vaccination, the age distribution of the daily new infected individuals changes over time, which further changes the age-specific risk of hospitalization. For this reason, and the fact that hospitalization risk changes for different virus variants [24,25], the modeled risk of hospitalization is time dependent.

### Hospital incidence data

Hospital incidence data for Norway was acquired by request from Norwegian Institute of Public Health. The data consist of weekly number of new hospital admission, for individuals with Covid-19 infection as main cause of hospitalization, for each of the 11 Norwegian regions.

Swedish data on hospital incidence was obtained from Socialstyrelsen [26]. They provide data on the weekly hospital incidence on a national level, as well as the number of currently treated individuals (occupied beds) on the county-level. County-level hospital incidence was not directly available. To approximate weekly hospital-incidence on the county-level, we distributed the national incidence to the counties based on the relative share of all individuals treated per county within the respective week.

### Likelihood of hospitalizations

The number of new hospitalizations, *H*_*w*,*r*_, in week *w* in region *r* was modeled by a Poisson distribution,


$$H_{w,r}\sim \text{Pois}(\sum_{t\in w}\sum_{j=0}^{m}\kappa_{t-j,r}u_{t-j,r}\tau_{j}),$$ (1)

where $\kappa_{t,r}$ denotes the probability of being hospitalized if infected at day *t* and belonging to region *r*; *m* is the maximum number of days from infection *t*o hospitalization and $\tau_{j}$ denotes the probability of going to hospital *j* days after infection, given that an infected individual goes to hospital. The quantity *u*_*t*,*r*_ is the number of new infections at day *t* in region *r* in our model, given by the sum of infections and imported cases


$$u_{t,r}=\frac{\beta_{t,r}}{N_{r}}I_{t,r}S_{t,r}+\frac{n_{\text{imp}}}{10^{\text{5}}}S_{t,r}.$$

If all infected individuals were hospitalized, then the expected number of individuals sent to hospital at day *t* will be the sum, over the preceding *m* days, of the number of new infections *j* days before day *t*, times the probability $\tau_{j}$ that those new infections are hospitalized after *j* days, i.e., at day *t*. Not all infected individuals need hospitaliza*t*ion, and we therefore multiply by $\kappa_{t-j,r}$, the probability that an individual infected at day *t* − *j* goes to hospital at day *t* (age-differences taken into account as explained below). The expected new hospitalizations in week *w* is then the sum over all days *t* in week *w*.

### Distribution of time between infection and hospitalization

The probability of being hospitalized *j* days after infection, given that an infected individual gets hospitalized, is denoted $\tau_{j}$. The time from infection to hospitalization consists of a pre-symptomatic period (time from infectiousness starts to symptom onset), followed by the period from symptom onset to hospital admission. Following the situational awareness and forecasting reports [2], Week 51, 29 December 2021, we assume that the pre-symptomatic period is exponentially distributed with mean 2 days, i.e., *Exp*(1/2). Norwegian data on time from symptom onset to hospitalization, provided by FHI on request, fits well with a negative binomial distribution, with mean and over-dispersion parameter changing with time. We assume these parameters are constant in time, and therefore resample data from the negative binomial for each time period, and estimate the mean (7.93) and over-dispersion parameter (6.21) of a negative binomial distribution from this data set. We assume that the length of the pre-symptomatic period and the symptomatic period are independent, and hence, $\tau_{j}$ is expressed as the convolution between the exponential distribution of the pre-symptomatic period and the negative binomial distribution of the time from symptom onset to hospitalization.

### Probability of hospitalization given infection

The probability of being hospitalized if SARS-CoV-2 infected is highly age dependent. The age distribution of the infected individuals in a region changes with age prioritized vaccination. The probability of hospitalization is also affected by the circulating virus variants. In the above likelihood of hospitalizations, the region specific parameter $\kappa_{t,r}$ is time dependent, but not age specific. We therefore need the time variation of the parameter to take into account both the changing age distribution of the infected individuals and the changing proportion of virus variants.

Let $\xi_{t,r}^{i}$ be the probability that an individual in region *r* infected at day *t* goes to hospital, given tha*t* the individual belongs to age group *i*. The probability that an individual infected at time *t* in region *r* is admitted to hospi*t*al is expressed as


$$\kappa_{t,r}=\sum_{\forall i}\xi_{t,r}^{i}\frac{S_{t,r}^{i}}{S_{t,r}},$$

where $S_{t,r}^{i}/S_{t,r}$ is the fraction of the susceptibles being in age group *i* in region *r* at day *t*. We have used values for $\xi_{t,r}^{i}$ for the “wild-type” variant as given in *t*he FHI modeling reports [2]. These probabilities are built on work by Salje et al. [27] and adjusted to fit a Norwegian population. However, vaccine data both for Norway and Sweden have different age groups, so we need to transform these probabilities to fit other age groups. A simple approach to this is to fit the exponential function *y* = *e*^(a+bx)^, where *x* denotes age, to the Norwegian specific probabilities. The estimated coefficients were *a* = −2.52 and *b* = 0.06. Probabilities for the new age groups were then inferred by choosing the fitted value *y* that corresponds to the middle age of each age group. See Fig 21 and Table 2 in S1 Appendix for age specific hospitalization risk for Norway and Sweden.

These risks of going to hospital for a given virus variant and age group is assumed constant throughout the pandemic, however, they change for different virus variants. The proportion of the different variant among the infectious individuals change over time, and hence do the risk of hospitalization. The risk of going to hospital if infected with the Alpha variant is estimated to be 1.9 times higher than if infected by the Wild-type [24,25]. The same holds for the Delta variant. This increase in hospitalization risk is assumed to be the same for all age groups. The overall risk of going to hospital, given infection, in terms of the proportion of the Alpha variant $x_{t,r}^{A}$ and Delta variant $x_{t,r}^{D}$ is then


$$\xi_{t,r}^{i}=\xi_{r}^{i,Wildtype}(\underset{\text{share of Wild-type}}{\underbrace{1-(x_{t,r}^{\alpha }+x_{t,r}^{\delta })}}+1.9\cdot \underset{\text{share of Alpha and Delta}}{\underbrace{\left(x_{t,r}^{A}+x_{t,r}^{D}\right)}}).$$

We only have access to variant data on a national level and therefore use the same data for all region within a country, despite the variants have probably spread differently in the different regions [21].

### Implementation

The meta-population and data model was implemented in Stan using the cmdstanr R-package to facilitate parameter estimation via Hamiltonian Monte Carlo. For a model fit to *n*^*reg*^ regions (11 for Norway, 21 for Sweden, and 2 for Germany) with 4 GCMR categories, 3 multiplicative factors on transmissibility (Temperature, and change in tranmissibility for Alpha and Delta VoC) in a period of *n*^*week*^ weeks (75 for Norway/Sweden, 44 for Germany) we estimated


$$\underset{\phi_{k,r}}{\underbrace{4*n^{reg}}}+\underset{\phi^{mult}}{\underbrace{3}}+\underset{\nu_{w,r}}{\underbrace{n^{reg}*n^{week}}}+\underset{\sigma_{r}^{\nu }}{\underbrace{n^{reg}}}$$

parameters and hyper-parameters. We used the Normal and (truncated) Half-normal priors as shown in Table 1:

**Table 1:** Priors used for model fitting.

Parameter	Prior	Description
$\phi_{k,r}$	$N_{\left[0,0.5\right]}^{+}(0,0.1)$	Region-spec. infections per GCMR category at BL activity
$\phi^{mult}$	*N*(0, 0.25)	Multipl. effects on transmissibility
$\nu_{w,r}$	$N_{\left[0,0.5\right]}^{+}(0,\sigma_{r}^{\nu })$	Weekly infections outside GCMR cat.
$\sigma_{r}^{\nu }$	*N*^+^(0, 0.1)	Region-specific SD of $\nu_{w,r}$

The data model for the region-specific weekly hospitalization counts consisted of a Poisson Likelihood as described above. The model was fitted using 4 independent chains with with 1000 samples (200 warm-up) each and convergence was monitored based on standard diagnostics ($\hat{R}<1.01$ for all parameters and visual investigation of trace plots). Source code including the Stan-model as well as the full analysis pipeline for the public data from Germany (based on the targets R-package) are provided in a Github repository https://github.com/FelixGuenther/nordic_behavior_public.

### Analysis for German regions

For fitting our model to two German regions we used 44 weekly data points on the reported 7-day hospitalization incidence provided by the Robert Koch-Institute from 1 March, 2021 to 27 December, 2022 for the federal states of Berlin and Bavaria [28]. These measurements contain all reported hospital cases from the last 7 days. We collected the publicly available GCMRs and obtained temperature data from the publicly available database of Deutscher Wetterdienst. As this analysis was focused on the year 2021 only, no data on vaccination or VoCs was required. Otherwise, model specification and fitting was performed as in the analysis for Norwegian and Swedish counties with one exception: as the population size of the German federal states is substantially larger compared to the Nordic counties (3.7 and 13.1 Million for Berlin/Bavaria) we reduced the daily importation of infectious individuals from 0.5/100,000 to 0.1/100,000 individuals to avoid an unrealistically high assumption on (absolute) imported cases, in particular during low-incidence periods.

## Supporting information

S1 Appendix Tables and figures supplementing the main text. (TEX)

## References

[1] Leung NHL. Transmissibility and transmission of respiratory viruses. Nat Rev Microbiol. 2021;19(8):528–45. doi: 10.1038/s41579-021-00535-6 33753932 PMC7982882

[2] FHI. Situational awareness and forecasting for Norway. 2021. https://www.fhi.no/sv/smittsomme-sykdommer/corona/koronavirus-modellering/

[3] Badr HS, Du H, Marshall M, Dong E, Squire MM, Gardner LM. Association between mobility patterns and COVID-19 transmission in the USA: a mathematical modelling study. Lancet Infect Dis. 2020;20(11):1247–54. doi: 10.1016/S1473-3099(20)30553-3 32621869 PMC7329287

[4] Aleta A, Martín-Corral D, Bakker MA, Pastore Y Piontti A, Ajelli M, Litvinova M, et al. Quantifying the importance and location of SARS-CoV-2 transmission events in large metropolitan areas. Proc Natl Acad Sci U S A. 2022;119(26):e2112182119. doi: 10.1073/pnas.2112182119 35696558 PMC9245708

[5] Chang S, Pierson E, Koh PW, Gerardin J, Redbird B, Grusky D, et al. Mobility network models of COVID-19 explain inequities and inform reopening. Nature. 2021;589(7840):82–7. doi: 10.1038/s41586-020-2923-3 33171481

[6] Fritz C, Kauermann G. On the interplay of regional mobility, social connectedness and the spread of COVID-19 in Germany. J R Stat Soc Ser A Stat Soc. 2022;185(1):400–24. doi: 10.1111/rssa.12753 34908652 PMC8662283

[7] LLC G. Google COVID-19 Community Mobility Reports. 2021. https://www.google.com/covid19/mobility/

[8] Cot C, Cacciapaglia G, Sannino F. Mining Google and Apple mobility data: temporal anatomy for COVID-19 social distancing. Sci Rep. 2021;11(1):4150. doi: 10.1038/s41598-021-83441-4 33602967 PMC7892828

[9] Nouvellet P, Bhatia S, Cori A, Ainslie KEC, Baguelin M, Bhatt S, et al. Reduction in mobility and COVID-19 transmission. Nat Commun. 2021;12(1):1090. doi: 10.1038/s41467-021-21358-2 33597546 PMC7889876

[10] Basellini U, Alburez-Gutierrez D, Del Fava E, Perrotta D, Bonetti M, Camarda CG, et al. Linking excess mortality to mobility data during the first wave of COVID-19 in England and Wales. SSM Popul Health. 2021;14:100799. doi: 10.1016/j.ssmph.2021.100799 33898726 PMC8058100

[11] Yilmazkuday H. Stay-at-home works to fight against COVID-19: International evidence from Google mobility data. J Hum Behav Soc Environ. 2021;31(1–4):210–20. doi: 10.1080/10911359.2020.1845903

[12] Deng Y, Lin H, He D. Trending on the use of Google mobility data in COVID-19 mathematical models. Adv Cont Discr Mod. 2024;21(2024). doi: 10.1186/s13662-024-03816-5

[13] Belsley DA. A Guide to using the collinearity diagnostics. Comput Sci Econ Manag. 1991;4(1):33–50. doi: 10.1007/bf00426854

[14] Stan Development Team. Stan Modeling Language Users Guide and Reference Manual, Version 2.31. 2022.

[15] Gabry J, Cesnovar R. cmdstanr: R Interface to ‘CmdStan’. 2021.

[16] Vehtari A, Gelman A, Simpson D, Carpenter B, Bürkner P-C. Rank-Normalization, Folding, and Localization: An Improved Rˆ for Assessing Convergence of MCMC (with Discussion). Bayesian Anal. 2021;16(2):667–718. doi: 10.1214/20-ba1221

[17] Violato C, Violato EM, Violato EM. Impact of the stringency of lockdown measures on covid-19: A theoretical model of a pandemic. PLoS One. 2021;16(10):e0258205. doi: 10.1371/journal.pone.0258205 34610042 PMC8491873

[18] Mossong J, Hens N, Jit M, Beutels P, Auranen K, Mikolajczyk R, et al. Social contacts and mixing patterns relevant to the spread of infectious diseases. PLoS Med. 2008;5(3):e74. doi: 10.1371/journal.pmed.0050074 18366252 PMC2270306

[19] Storvik G, Diz-Lois Palomares A, Engebretsen S, Rø GØI, Engø-Monsen K, Kristoffersen AB, et al. A sequential Monte Carlo approach to estimate a time-varying reproduction number in infectious disease models: the Covid-19 case. J R Stat Soc Ser A Stat Soc. 2023;186(4):616–32. doi: 10.1093/jrsssa/qnad043

[20] Aktay A, Bavadekar S, Cossoul G, Davis J, Desfontaines D, Fabrikant A, et al. Google COVID-19 Community Mobility Reports: Anonymization Process Description (version 1.1). 2020. Available from: https://arxiv.org/abs/2004.04145

[21] FHI. Weekly Covid-19 reports, FHI. 2021. https://www.fhi.no/en/publ/2020/weekly-reports-for-coronavirus-og-covid-19/

[22] Fohm. Statistik om SARS-CoV-2 virusvarianter av särskild betydelse. 2021. https://www.folkhalsomyndigheten.se/smittskydd-beredskap/utbrott/aktuella-utbrott/covid-19/statistik-och-analyser/sars-cov-2-virusvarianter-av-sarskild-betydelse/

[23] Folkehelseinstituttet. GitHub respository of The National Institute of Public Health. 2021. https://github.com/folkehelseinstituttet/surveillance_data

[24] Veneti L, Seppälä E, Larsdatter Storm M, Valcarcel Salamanca B, Alnes Buanes E, Aasand N, et al. Increased risk of hospitalisation and intensive care admission associated with reported cases of SARS-CoV-2 variants B.1.1.7 and B.1.351 in Norway, December 2020 –May 2021. PLOS ONE. 2021;16(10):1–12. doi: 10.1371/journal.pone.0258513 PMC8504717 34634066

[25] Veneti L, Valcarcel Salamanca B, Seppälä E, Starrfelt J, Storm ML, Bragstad K, et al. No difference in risk of hospitalization between reported cases of the SARS-CoV-2 Delta variant and Alpha variant in Norway. Int J Infect Dis. 2022;115:178–84. doi: 10.1016/j.ijid.2021.12.321 34902584 PMC8664610

[26] Socialstyrelsen. Swedish covid-19 hospitalization data from Socialstyrelsen, dataset: Nyinskrivningar och avlidna under pandemin. 2022. https://www.socialstyrelsen.se/statistik-och-data/statistik/statistik-om-covid-19/

[27] Salje H, Kiem CT, Lefrancq N, Courtejoie N, Bosetti P, Paireau J, et al. Estimating the burden of SARS-CoV-2 in France. Science. 2020;369(6500):208–11. doi: 10.1126/science.abc3517 32404476 PMC7223792

[28] Robert Koch-Institut. COVID-19-Hospitalisierungen in Deutschland. 2023. https://github.com/robert-koch-institut/COVID-19-Hospitalisierungen_in_Deutschland/

